# Emotional Contagion Scale and Mental Health Status during the First Wave of COVID-19 Pandemic, National Assessment

**DOI:** 10.2174/17450179-v18-e2208200

**Published:** 2022-08-29

**Authors:** Samar A. Amer, Eman Elsayed Abd-Ellatif, Peter Hughes, Hadi Mahdi Ghazai Al-Enazi, Ahmed AlHasan, Mostafa A. Amer, Asma Faleh Alruhaimi, Mohamed Elsayed

**Affiliations:** 1 Department of Public Health and Community Medicine, Faculty of Medicine, Zagazig University, Zagazig 44519, Egypt; 2 Member at the Royal College of General Practitioners (INT), London, UK; 3 Department of Public Health and Community Medicine, Faculty of Medicine, Mansoura University, Mansoura, Egypt; 4 Springfield University Hospital, London, UK; 5 Nursing Specialty, Director of the Anti-Smoking Program in Al-Qassim, Ministry of Health (MOH), Saudi Arabia, SA; 6 MD, General Directorate of Medical Services, Ministry of Defense, SA; 7 Faculty of Medicine, Zagazig University, Zagazig 44519, Egypt; 8 Department of Epidemiology, General Directorate of Health Programs and Chronic Disease, MOH, SA; 9 Department of Psychiatry and Psychotherapy III, University of Ulm, Leimgrubenweg 12-14, 89075, Ulm, Germany; 10 Department of Psychiatry, School of Medicine and Health Science, Carl von Ossietzky University Oldenburg, Oldenburg, Germany

**Keywords:** Emotional contagion scale, Mental health, Anxiety, Depression, COVID-19, Preventive measurements

## Abstract

**Background:**

This great pandemic of COVID-19 has been a unique stressor that affected all communities in 2020. This study aims to examine the prevalence of anxiety and depression due to the COVID-19 pandemic in Saudi Arabia and to study the emotional cognition scale in the Kingdom of Saudi Arabia (KSA) in relation to the COVID-19 pandemic.

**Methodology:**

A descriptive cross-sectional study was conducted on 857 inhabitants randomly selected from the 13 administrative regions of Saudi Arabia, using a validated self-administrated questionnaire comprising six sections. The collected data were summarized and analyzed.

**Results:**

Among the majority of the studied participants, 377 (44.0%) were aged from 35 to less than 50 y. There were 489 (57.1%) females and 368 (42.9%) males, 616 (71.9%) Saudi nationals, 715 (83.4%) university-educated or postgraduate, 619 (72.2%) unmarried and 238 (27.8%) married, and 663 (77.4%) living in areas under partial lockdown. The resultant elevated total depression score was statistically significant (p<0.05) for the following: participants younger than 35y, females, Saudis, those with lower education levels, those who were married, students, those with work suspension during the COVID-19 pandemic, and amongst those who experienced complete lockdown. Among the majority of the studied participants, 355 (41.2%) showed mild depression, and 281(32.6) showed moderate anxiety, and were in the growth zone. In addition, the elevated total anxiety score was statistically significant (p<0.05) amongst the following; younger participants, females, Saudi nationals, those with lower educational levels, those who were unmarried, students, those with telework, and those with no curfew.

**Conclusion:**

The adverse mental health effects were more prevalent among particular groups of the population, such as females, adults under 35 years old, students, those with lower educational attainments, and those suffering from chronic illnesses. Anxiety was significantly correlated with depression. The practice of preventive measures, e.g., wearing masks, and social distancing to prevent the spread of COVID-19, may have had psychological benefits during the pandemic.

**Summary:**

We assessed the mental health status in Saudi Arabia during the first wave of the COVID-19 pandemic. Mild depression and moderate anxiety were prevalent problems, with many determinants and interrelations. Fear was the most infectious emotion, while happiness was the highest.

## 1 INTRODUCTION

A few months after the detection of the Severe Acute Respiratory Syndrome Coronavirus-2 (SARS-CoV-2), the World Health Organization (WHO) announced that COVID-19 disease was a worldwide pandemic due to the high number of international cases [1, 2]. The pandemic of COVID-19 and its resulting lockdown regulations affected all, especially vulnerable groups [3] in Saudi Arabia (SA), as well as globally [4].

The psychological reactions to the pandemic were seen not only in healthcare workers but also in the general population [5]. The lockdown measures themselves triggered adverse psychological reactions [6]. Certain factors provoked those psychological problems, such as the extent of exposure to COVID-19 patients [7], quarantine measures [8], social withdrawal [9], as well as anxiety-provoking information shown on the various media outlets [10].

The main psychological issues that resulted were stress, depression, anxiety, feelings of frustration, and worries about the future [5, 6, 11]. Other studies among the general population reported a high prevalence of emotional disturbances, including mood swings [12] and irritability [13], post-traumatic stress symptoms [14], insomnia [15], and anger [16].

It has been seen that emotional distress during the COVID-19 outbreak can result in maladaptive behaviors [17]. Especially when the coping strategies of the individuals are overwhelmed. Maladaptive behaviors are behaviors that interfere with daily activities or the ability to adjust to different settings, ranging from minor behaviors (*i.e*., nail-biting, difficulty separating) to more severe behaviours (*i.e*., self-injurious or over-sexualized behaviors) [18]. The stress response can lead to non-adherence to COVID-19 preventative measures such as social distancing and mask wearing. It is important to evaluate the correlation between the degree of psychological disturbance due to the COVID-19 pandemic and the degree of the practice of preventive measures to limit the spread of the COVID-19 infection.

A comprehensive assessment and analysis of the different age groups suffering from anxiety and depression during the COVID-19 pandemic have been explored in previous research [19]. However, this has been in other regions with a different cultural background [19-23]. Therefore, the assessment of different age groups and their correlation with psychological distress among community members in Saudi Arabia during the COVID-19 outbreak is clinically important.

In this study, we specifically looked specifically at an assessment of the knowledge of the community regarding hygienic measures to reduce the spread of COVID-19 infection and the psychological benefit of this.

The following was the study's goal:

To examine the prevalence of anxiety and depression due to the COVID-19 pandemic, to study the emotional Cognition scale and how this may be of use, and to explore how psychological distress can inversely relate to COVID-19 preventative measures.

## 2 MATERIALS AND METHODS

### 2.1 Study Design and Participants

This cross-sectional online survey was designed for internet users. The target population was adults between the ages of 18 and 70 years old, both Saudi and non-Saudi, living in Saudi Arabia (SA) during the first wave of the COVID-19 pandemic in 2020. Exclusion criteria included refusal to participate in the study, not using the internet, being illiterate, and being under the age of 18 or over the age of 70. Those with complicated medical or mental health conditions such as psychosis were excluded.

The first COVID-19 case in KSA was detected on March 2, 2020. At the beginning of this study, there were 65077 confirmed COVID-19 cases and 400 deaths all over the Kingdom, mainly in the Eastern Region, Mecca, and Riyadh. The study was conducted from May 2020 to July 2020 [24].

### 2.2 The Sample Size

As a cross section study, the sample size was estimated according to the following equation: The sample size (n) is calculated according to the formula: n = z^2^ * p * (1 - p) / d^2^. Where: z = 1.96 for a confidence level (α) of 95%, p = proportion (expressed as a decimal), d = margin of error [25].

There is limited data about the prevalence of mental or emotional health disorders in Saudi Arabia during COVID-19. A previous study showed [26]. The prevalence rate for significant depression was 42.9% with a 95% confidence level and 54.9% with an 80% power of the study. With this information, the calculated sample size was 420 participants. In this study, we took double this figure, with an N of 857.

### 2.3 Sampling Method

Using a multi-stage sampling method. The sample was collected from all the administrative regions and weighted per proportion of population density, and the percent of urban to rural in KSA was found to be 213 (24.9%). Riyadh, 218 (25.5%) Al-Mokaramah, 56 (6.5%); Al-Momawarah, 39 (4.5%); Al-Qassem, 125 (14.5%); and the Eastern Region, 62 (7.3%). Asser, 26 (3.0%) Tabouk, 20 (2.3%) 10 (1.2) Northern borders, 44 (5.1) Gazan, 16 (1.9%) Najran, 14 (1.6%) Al Baha, 14 (1.6%), Al Jouf. Then data from each administrative region was collected (90% from inside the city, and 10% from outside the city) [27].

### 2.4 Data Collection

The data was collected through an online self-administered, structured questionnaire. Participants completed and submitted the questionnaire after approval of participation in the study. The participants gave informed consent. The questionnaire was distributed through the most common platforms in SA (Twitter, Snap Chat, and WhatsApp groups). In addition, and to have a representative sample, we used the official platforms of every 20 Health regions all over SA.

To increase the response rate, reminder messages were sent every week until the target response figures were met. Pilot testing was done and involved 15 participants in order to ensure clarity of the questionnaire, and the results of the pilot were not included in the study. Cronbach alpha was calculated to be 0.87%.

#### 2.4.1 The Data Collection Tool

The questionnaire was composed of six main sections as follows;

(1) Age, gender, nationality, level of education, residence, marital status, occupation, employment status, experience with COVID curfew types, smoking history, and history of chronic diseases.

(2) Current status of COVID-19 contact with any confirmed case, having any suspicious symptoms, COVID-19 infection, and recovery from COVID-19

(3) The sources of information about COVID-19 included television, social media, radio, public health messaging, etc.

(4) Hygiene precautionary measures knowledge about sneezing etiquette and hygienic hand washing techniques.

The practice of preventive measurements during the previous three weeks (outdoors) was assessed through 9 questions with a Likert score (No, and rare = 1/ Sometimes and often= 2/ Always = 3).

(5) Evaluation of mental health status.

##### 2.4.1.1 Patient Health Questionnaire-9 scale (PHQ-9)

 We screened for depression in a “first-step” approach and asked about the frequency of depressed mood and anhedonia over the previous two weeks. The Likert scale for depression was (0-not at all/1-several days/two—more than half the days/3-nearly every day). The degree of severity based on the total score was (0-4 = Minimal or none/5-9= Mild/10-14 = Moderate/15-19 = moderately severe/20-27 = severe) [14, 28].

##### 2.4.1.2 Generalized Anxiety Disorder Assessment (GAD-7) (GAD 7)

This is a very brief and easy to perform initial screening tool for generalized anxiety disorder through a Likert scale of 0-not at all/1-several days /2—more than half days/3-nearly every day [29].

##### 2.4.1.3 Emotional Cognition Scale (EC scale)

This is a 15-item index, it examines mimetic tendency to five basic emotions (love, happiness, fear, anger, and sadness). It aims to measure individual differences in susceptibility to picking up the emotions of other individuals and then experiencing that same feeling [30, 31].

(6) The Classification of Participants during the COVID-19 pandemic;

We used the British Colombia COVID-19 online Self-Assessment Tool that was developed by the British Colombia (BC) Ministry of Health. This describes different zones of fear, learning, and growth. The tool gives guidance on how to change zones based on the symptoms experienced [32].

#### 2.4.2 Growth Zone

I practice quietude, patience, relationships, and creativity.

Looking for new ways to disease.

I thank and appreciate others.

Think about others and help them.

Do your best to support others.

#### 2.4.3 Learning Zone

I evaluate information before spreading something false

I identify my emotions

I stop compulsively consuming what hurts me, from food to news

I start to give up what I can’t control

#### 2.4.4 Fear Zone

I complain frequently

I spread emotions related to fear, and anger

I grab food, toilet paper and medications that I don’t need

### 2.5 Statistical Analysis

The data were analyzed using SPSS version 25 and a level of significance (p<0.05). Qualitative data were presented as frequency and percent, while quantitative data were presented as mean± SD, median, and range. Chi2 test was used to test the association between categorical variables. T-test, ANOVA (Analysis of Variance), Kruskal Wallis test were used to test the association between quantitative variables. Pearson's correlation coefficient (r) was used to test the association between two continuous variables.

### 2.6 Ethical Issues

Ethics Committee of the King Fahad City, The Saudi ministry of health IRP (Exempt type) H-01-R-012, approved the study methodology. All participants provided electronic informed written consent after clarification of the goals, data confidentiality, voluntary participation, and withdrawal.

## 3 RESULTS

### 3.1 The Demographic Characteristics

Among the studied participants, 377(44.0%) were aged between 35 and 50 years old. There were 489(57.1) females and males (42.9%). There were 616(71.9%) Saudi nationals. 715(83.4%) were university-educated or postgraduate. 619(72.2%) were unmarried. 768(89.6%) were urban. 497 (58.0%) were working in the medical field, 63(77.4%) were living in areas with a partial lockdown at the time of the study. 381(44.5) were working as before the COVID-19 pandemic. In terms of work status, nearly half of the 381 participants (44.5%) reported that their work status had not changed because of the COVID-19 pandemic. Almost three-quarters of the participants (663 or 77.4 percent) were subjected to a partial curfew (Table **1**).

### 3.2 The Relationship between Total Depression Score and Demographic Characteristics

The total depression score was statistically significant (p<0.05) in a number of areas. The results were significant, with higher depression among participants less than 35y - 7.5(8.3 ±5.5) (0-20), females 6(7.2 ±6.4), Saudi nationals, those with lower educational levels, those who only read and write n 13(13 ±5.7)(9-17), married 8(8.6 ±6.7), students 8(8.6 ±6.7), those with work suspended 6(7.5 ±6.3) and complete lockdown 10.5(11.1 ±6.9) (Table **1**).

### 3.3 The Relationship between Total Anxiety Score and Demographic Characteristics

The total anxiety score was statistically significant (p<0.05) with a higher score among participants in the age group between 20 to 30 years {4(5.1 ±5.50(0-21)}, females 3(4.8+_5.3)(0-21), Saudis 2(4.2 ±4.9)(0-21), lower education levels (read and write) 10.5(10.6 ±10.5), and unmarried 4(5.5 ±4.5), students 5(6.3 ±5.6), with Tele work 3(4.5 ±4.9) and with no curfew 7(7.8 ±3.9) (Table **1**).

If there was a history of contact with someone with confirmed or suspected symptoms of COVID 19, there were significantly higher rates of depression and anxiety scores (Table **2**). The main sources of information about COVID-19 among the studied participants were Scientific and official websites (83.3%), Social media (38.9%), Mass media (36.3%), and other Websites (19.4%).

### 3.4 The Relationship between the Regions and the Total Anxiety, and Depression Scores

There were statistically significant differences between different health regions in their total scores of anxiety and depression. The total scores were significantly higher in Al Qureat 23.1 ±2.4, Tabouk 23 ±2.9, Northern border 21.4±3.7, and Hael 21.  ±7.0 and the lowest at Hafr El Batten. The total depression scale was significantly higher in Hael 19.5 ±0.7, Mecca 12(11.6 ±6.9), Asser 12(10.3 ±6.9), Riyadh 11.5(10.9 ±6.7), and lowest were in Al Qureat 0 (3.2 ±5.3). The total anxiety scores were significantly higher in Hael 8.5(2.5 ±2.1), Riyadh 6(7.6 ±6.1), and Mecca 6(7.6±5.8), and the lowest were the Northern Border and AlQureat (Table **3**).

### 3.5 Level of Knowledge, and Practice of Preventive Measurements

The majority of the studied participants (> 94%) stated good knowledge about the practice of sneezing etiquette and hygienic hand washing. More than three-quarters of study participants (> 70%) mentioned the good practice of washing hands with soap and water or alcohol, keeping a safe social distance, sterilizing surfaces, wearing face masks, sanitizing purchases, and staying at home. The median of the precaution preventive COVID-19 Practice score was 20 with a range of (9-27) while (mean±SD) was 19.7±3.9 (Table **4**).

### 3.6 The Level Of Anxiety, Depression, And Their Interrelations Among The Studied Participants

The total anxiety GAD-7 scores were as follows; Median (mean ±SD) (minimum-maximum) were two (4.01±4.9) (0-21) respectively. Nearly 60% of the participants in the study had a GAD-7 score of mild to moderate anxiety, while 30.2 percent and 9.8 percent had none and severe anxiety, respectively (Table **5**). The total score of depression (PHQ-9) was median (mean± SD) (minimum-maximum) was 5(6.4±6.0) (0-27) respectively. The prevalence of depression by symptom severity by the PHQ-9 was that more than 60% of study participants had mild to moderate depression, while 30.2 percent and 9.8 percent had none and severe anxiety, respectively (Table **5**).

There was a positively strong significant correlation between anxiety and depression (r=0.79), with (p< 0.0001). Respondents with higher levels of anxiety were found to be more likely to use preventive measures(r=0.01) with a non-significant association p-value =0.73. Preventive measures use is inversely related to depression levels(r=-0.02) with a non-significant association p-value =0.49 (Table **6**).

### 3.7 The Main Participants’ Perception And Self-Assessment Towards The COVID-19 Pandemic

In terms of the British Colombia COVID-19 online Self-Assessment Tool, Table **7** shows that more than 89 percent of participants in the growth and learning zones answered yes. Furthermore, in the fear zone, more than 79 percent said no.

Table **8** showed that the main participants’ perceptions or feeling towards COVID_19 were as follows (75.1%) fear that they would infect their family, (72.7%) that the courses of the disease are in the hand of God (72.7%), and fear of COVID 19 complications as they have risk factors (25.9%).

### 3.8 The Emotional Contagion Scale Among The Studied Participants

The Emotional Contagion Scale measures the individual differences in susceptibility to catching the emotions of other individuals. Emotional contagion describes a certain sensitivity to the emotions of others and induces the individual to unintentionally 'catch' those emotions from mere exposure to others' behavior. The EC Scale shows that the highest measured emotional arousal, the congruence of emotional stimulus and response in KSA during the first wave of COVID-19 pandemic were at the following domains in descending orders; (happiness, love, Anger, sadness than fear Table **9**. So we say by this that fear can be the least infectious emotion while happiness is the highest.

## 4 DISCUSSION

During an outbreak of infectious disease, the psychological reactions of the population play a critical role in shaping both the spread of the disease, the pattern of mortalities, and the occurrence of emotional distress and social disorder during and after the outbreak. It is well understood that psychological factors influence both adherence to public health measures (such as vaccination) and how people cope with the threat of infection and the resulting losses [33]. Psychological responses to pandemics include maladaptive behaviors, emotional distress, and defensive responses [34]. People who are predisposed to mental illnesses are especially vulnerable [33].

The main sources of information about COVID-19 among the studied participants were scientific and official websites (83.3%), social media (38.9%), mass media (36.3%), and other websites (19.4%). Furthermore, the results of this study were higher than those reported in an Ethiopian study, where the internet (84%) and TV (44%), while among Egyptians, the main sources of information were social media (64.5%), followed by international and governmental official websites (12.8%), and inconsistent with the results of a study in Syria were TV (66.4%), followed by government officials (38.7%), Facebook (34.8%), health workers (31.4%), websites (23.8%), and family and friends (43.3%) [35, 36]. The scientific and official websites proved to be helpful during the COVID-19 compulsory lockdown in KSA.

As regards the depressive symptoms associated with the pandemic, more than half of the participants (52.1%) were seen to suffer from moderate to severe depression, and 128 (21.9%) participants displayed severe depressive symptoms. The findings from KSA are higher than what was reported during the early stage of the COVID-19 outbreak in China (January–February 2020). In this case, of China, 54% rated the psychological impact of the COVID-19 outbreak symptoms as moderate to severe, and 17% reported moderate-to-severe depressive symptoms [37].

While nearly one-third of those polled (28.6%) in KSA experienced mild to moderate anxiety, these findings are higher than those reported in a previous Saudi Arabian study during the pre-COVID-19 pandemic. One hundred and four (17.6%) displayed moderate-severe anxiety [38]. 54% rated the psychological impact of the COVID-19 outbreak as moderate to severe anxiety symptoms, while 29% reported moderate to severe anxiety symptoms [37], but it is still not understood the statement-what is the psychological impact? Is it a generic 54% reported psychological effects of the COVID-19 pandemic? Inconsistencies in statistics in this study in KSA could be attributed to the country's social, economic, and political structures, as well as disease-related factors. Different levels of anxiety, depression, and safety precautions may be caused by cultural, social, educational, and disease-related factors in different parts of Saudi Arabia.

According to our sociodemographic data, females experienced a greater psychological impact from the pandemic, as well as higher levels of stress, anxiety, and depression. This finding is consistent with previous large epidemiological studies that found women to be at a higher risk of depression [39], and the literature shows that women in KSA have poorer mental health and wellbeing than men [40].

### 4.1 The findings of this Study Revealed That Older Participants Had Better Mental Health

The age group of 35–50 years old had the highest prevalence of mental disorders. This finding is consistent with the findings of an Iranian study conducted during the COVID-19 pandemic [11], where older people had better mental health. This result, however, contradicts the findings of previous mental health surveys conducted under normal conditions [41]. This resilience among older participants may be due to the interaction of many internal factors; for example, physical health, cognitive capacity, biological response to stress, and personality traits, along with other external resources;

These results are very important for public health because of the current situation, caused by COVID-19 and the lockdown. The complete curfew was statistically significantly associated with the highest median total anxiety score of 6 and the total depression score of 12. These results are very important for public health because of the current situation, caused by COVID-19 and the lockdown that followed [42].

This puts older people at a high risk of stress. Social functionality is an important element of resilience to stress and depression. Older adults experienced significantly during the COVID-19 pandemic due to less social contact with relatives, friends, and healthcare workers; poor access to care; an interruption of daily routine activities; and poor social communication. Moreover, they are at the highest risk of death if infected. Thus, all factors that could determine resilience to stress should be investigated and studied to provide useful plans for prevention in the future [43].

Students were also found to be psychologically affected by the pandemic, with higher levels of anxiety and depression. This finding could be explained by the closure of all schools at all levels. The uncertainty and potential negative impact on academic progression may have a negative impact on student’s mental health.

The public with reading and writing skills or primary education had a higher likelihood of depression and anxiety during the pandemic, according to this study. To assist those with a lower educational background during the pandemic, local agencies must provide information in an easy-to-read, diagrammatic, or audio format using simple language [44].

We found a higher risk of mental health disorders among participants whose work was suspended or who were under a complete curfew. This is matched by an Italian study. This could be explained by COVID-19-related working difficulties [5].

According to a systematic review of the impact of the COVID-19 pandemic on mental health in the general population worldwide, anxiety is frequently comorbid with depression. Some predictors of anxiety and depression symptoms include a younger age group (40 years), lower education levels, poor self-rated health, loneliness, female gender, divorced or widowed status, quarantine status, worry about being infected, property damage, history of mental health issues or medical problems, presence of chronic illness, and living in urban areas [45]. These findings agree with ours.

In this study, only 82 (9.6 percent) and 51 (6.0%) of the respondents had direct or indirect contact with people who had confirmed or suspected COVID-19. These findings are higher than those reported in the China study, where 1 percent had a history of contact with COVID-19 cases [39]. The first COVID-19 case in KSA was detected on March 2, 2020. At the beginning of this study, there were 65077 confirmed COVID-19 cases and 400 deaths all over the Kingdom, mainly in the Eastern Region, Mecca, and Riyadh. The study was conducted from May 2020 to July 2020 [24].

During the COVID-19 pandemic, the Internet (scientific and official websites) was the main public source of health information about COVID-19 (83.3%). This finding is lower than that reported during the early stages of the COVID-19 epidemic in China, where the Internet (93.5%) was the primary health information channel for the public [37].

The vast majority of respondents (about 94%) reported expert knowledge of both sneezing etiquette and hygienic hand washing. More than three-quarters of study participants (more than 70%) reported washing their hands with soap and water or alcohol, keeping a safe distance, sterilizing surfaces, wearing facemasks, sanitizing purchases, and staying at home. This is higher than the results from China, where more than half of the people surveyed washed their hands with soap after touching something contaminated, covered their mouths when they coughed or sneezed, and wore masks as a precaution whether they had symptoms or not [38].

Our findings suggest that anxiety is significantly correlated with depression in relation to COVID-19. The significantly higher prevalence of reported adverse mental health conditions associated with the COVID-19 pandemic highlights the pandemic's broad impact. It highlights the need to prevent and treat these conditions and identify vulnerable groups. The identification of populations at high risk for psychological distress and unhealthy coping can help shape policies to address health inequity, such as increasing access to resources for clinical diagnosis and treatment options. Increased use of telehealth, which is a good way to treat mental health problems like depression and anxiety, may reduce the mental health effects of COVID-19 [46, 47].

The practice of preventive measures is inversely related to depression levels. Specific precautionary measures, such as practicing hand hygiene and wearing masks regardless of symptoms, were associated with lower levels of depression, anxiety, and stress [36]. In agreement with what has been reported in the United States, the use of proactive precautionary measures such as avoiding people who cough, unnecessary travel, and the use of public transportation or public places [48], was associated with lower COVID-related anxiety even if people experienced isolation. Having more close or meaningful relationships may be more protective than just having more interactions with others, as the quality of the relationship is key rather than the number of social connections [49].

This finding was inconsistent with the Chinese study [37]. This discrepancy may be explained by the 2003 SARS-CoV-I epidemic in China, where the general public's perception of precautionary measures was changed, leading to a positive effect on the initial psychological responses to the COVID-19 pandemic by giving respondents confidence and a sense of control in prevention. This reason could explain the difference between the Saudis and the Chinese population.

Following the British Colombia COVID-19 online Self-Assessment Tool results, during the first wave, the majority of the studied participants were in the growth and learning zones, and only a little less than 15% were in the fear zone. This can be explained by the fact that 83.3% of the studied participants got information from trusted scientific and official websites.

As regards the ECS, happiness was the emotion of high cognition among the studied participants, in agreement with the recently published report by the UN Sustainable Development Solutions Network about the World Happiness Rank of 95 countries in 2021 during the COVID-19 pandemic, which reported that KSA was the 1st Arabic country and the 21st global country according to the happiest index [50].

### 4.2 Strength

A relatively large representative sample of the 13 geographic health regions from all over the KSA. This study included 24 nationalities. We conducted a complete mental and emotional cognition assessment through a detailed questionnaire using four validated scales and scores.

### 4.3 Limitations

being a cross-sectional study. The majority of participants who were working in the medical field may be a bias, but they were the most active users of websites.

## CONCLUSION

There is a high prevalence of reported adverse mental health among populations such as females, people in the age group of 35, students, those of lower educational levels, and those suffering from chronic illnesses. Anxiety is significantly correlated with depression in this study in relation to COVID-19 infection. The precautionary measures taken to prevent the spread of COVID-19 may have led to psychological benefits during the pandemic. The EC Scale provides that the highest measured emotional arousal and the congruence of emotional stimulus and response in KSA during the first wave of the COVID-19 pandemic were happiness and love—that is, happiness was more readily spread between people.

## RECOMMENDATIONS

We recommend that you: 1) Involve mental health care and supportive teams in the management plan of an outbreak or pandemic in preparedness and at all stages. 2). Integration of mental health care in primary care settings is essential. 3) Special attention and targeted mental health programs for vulnerable or at-risk groups, such as students, women, and frontline health care providers. 4) Easily accessible and understandable official sources of information.

## Figures and Tables

**Table 1:** Sociodemographic data and its relationship with depression and anxiety scores.

-	**Total** **F (%)**	**Depression Score** **Median (Mean ±SD) (range)**	**Anxiety Score** **Median (Mean ±SD)(range)**
**Age groups** **<20 year** **20-<35y** **35-<50y** **50-<65y** **65 or more**	12(1.4)360(42.0)377(44.0)105(12.3)3(0.4)	7.5(8.3±5.5) (0-29)7(8.0±6.5) (0-27)4 (5.5±5.2) (0-27)3(4.1±4.8) (0-18)1(0-3)	3(5.3±5.4) (0-16)4(5.1±5.50(0-21)2(3.5±4.6) (0-21)1(1.9±2.9) (0-16)3(3.3±_2.5)(1-6)
**P**		0.00*	0.04*
**Sex** **Males** **Females**	368(42.9)489(57.1)	4(5.4±5.4)6(7.2±6.4)	1(2.9±4.1) (0-21)3(4.8±5.3)(0-21)
**P**		0.00*	0.00*
**Nationality** **Saudi** **Non -Saudi**	616(71.9)241(28.1)	5(6.7±5.9)4(5.7±6.1)	2(4.2±4.9) (0-21)2(3.7±4.9)(0-21)
**P**		0.01*	0.03*
**Level of education** **Read and write/primary** **Preparatory, secondary** **University and above**	2(0.2)140(16.3)715(83.4)	13(13±5.7) (9-17)5(6.3±5.9) (0-27)5(6.4±6.1)(0-27)	10.5(10.6±10.5)2(4.1±4.9) (0-21)2(3.9±4.9)(0-21)
**P**		0.00* 0.00*	
**Marital status** **Married** **Unmarried**	238(27.8)619(72.2)	8(8.6±6.7)4(5.6±5.5)	2(3.5±4.6)4(5.5±4.5)
**P**	0.00* 0.04*		
**Residence** **Outside the city** **Inside the city**	89(10.4)768(89.6)	4(5.7±5.8)5(6.5±6.0)	2(3.4±4.6)2(4.1±4.5)
**P**		0.24	0.10
**Having chronic diseases** **No** **Yes**	655(76.4)202(23.6)	10(9.4±6.7)8(8.7±7.1)	4(6.1±5.9)4(5. ±5.9)
**P**	0.19 0.24		
**The occupation** **No** **Student** **In the medical field** **Outside the medical field**	140(16.3)48(5.6)497(58.0)172(20.1)	6(6.9±6)8(8.6±6.7)4(5.9±6.1)5(6.5±5.4)	3(4.7±5.2)5(6.3±5.6)2(3.8±5.0)2(3.4±3.9)
**P**		0.00*	0.01*
**Working status** **As it is** **Telework (working remotely)** **Decreased working hours** **Work suspension**	381(44.5)199(23.2)191(22.3)86(10.0)	5(6.2±6.1)6(6.9±6.1)4(5.8±5.6)6(7.5±6.3)	2(3.9±4.9)3(4.5±_4.9)2(3.5±4.7)3(4.8±5.5)
**P**		0.00*	0.04*
**The curfew types** **No** **Partial** **Complete**	6(0.7)663(77.4)188(21.9)	10.5(11.1±6.2)8(8.7±6.7)12(11.5±.9)	7(7.8±3.9)4(5.6±5.4)6(7.8_±3.9)
**P**		0.00*	0.03*

Notes: *p<0.05 there was a statistically significant relationship.

**Table 2:** The status as regards the exposure to COVID-19.

-	**Total** **F (%)**	**Depression Score** **Median (Mean ±_SD)**	**Anxiety Score** **Median (Mean ±SD)**
**Contact with confirmed case** **Yes** **No**	51(6.0)810(94.0)	5(6.4±5.9)5(7.3±7.2)	2(3.9±4.9)4(5.2±5.7)
**P**		0.18	0.00*
** Contact with confirmed cases ** **Yes** **No**	82(9.6)779(90.5)	6(8.2±7.6)5(6.2±5.8)	3.5(5.3±5.6)2(3.9±4.8)
**P**		0.03*	0.00*
**Having suspicious symptoms #** **Yes** **No**	10(1.2)851(98.8)	15.5(15.2±8.9)5(6.3±5.9)	8(10.4±7.6)2(3.9±4.9)
**P**		0.00*	0.00*
**COVID-19 case (infected)** **Yes** **No**	3(0.4)858(99.7)	0(4.3±7.5) (0-12)5(6.4±6.0)(0-27)	2(1.3±1.2)2(4.02±4.9)
**P**		0.00*	0.00*
**Recovered from COVID-19** **Yes** **No**	231(27.0)630(73.2)	5(5.9±5.4)5(6.6±6.2)	2(3.4±4.4)2(4.2±5.1)
**P**		0.11	0.34
**Seeing COVID-19 cases on social media** **Yes** **No**	554(64.6)307(35.7)	6(6.9±6.1)4(5.5±5.7)	3(4.3±5.01)2(3.4±4.7)
**P**		0.08	0.06

Notes: * There are statistically significant differences (p <0.05).

#Fever, and /or dry cough, and /or difficult breathing).

**Table 3:** The total scores of anxiety, depression, and practice among different health regions.

**The Health Regions**	**The Total Scores**		
	**Anxiety** **Median (Mean±SD)**	**Depression** **Median (Mean±SD)**	**Practice** **Mean±SD**
**Al Ahsa**	5(6.1±6.3)	7(9.3±11.2)	19±2.2
**Al Jouf**	1(3.2±5.9)	4(5.3±6.1)	17.5±4.7
**Northern border**	0(2.6±4.8)	6(9.2±7.6)	21.4±3.7
**Riyadh**	6(7.6±6.1)	11.5(10.9±6.7)	19.3±3.9
**Eastern region**	5(5.9±5.4)	10(9.9±5.2)	19.7±2.9
**Al Taif**	2(4.9±4.8)	8(7.7±6.2)	20.9±3.6
**Al Qureat**	0(2.9±5)	0(3.2±5.3)	23.1±2.4
**Al Qassem**	2(5±5.0)	6(7.8±6.6)	19.7±4.1
**Al- Madinah al Monawarh**	2(5.8±6.9)	8(8.**5**±**7**.1)	19.9±2.9
**Tabouk**	6(6.0±5.7)	8.5(8.5±2.1)	23±2.9
**Gazan**			16.8±4.1
**Jeddah**	6(6.6±5.8)	10(10.6±4.9)	20.03±3.1
**Hael**	8.5(2.5±2.1)	19.5±0.7	21. ±7.0
**Hafr El Baten**	2	10(7.3±4.6)	16.7±6.4
**Asser**	5(7±7.4)	12(10.3±6.9)	17.9±5.3
**Makkah**	6(7.6±5.8)	12(11.6±6.9)	20.7±4.2
**Yanbou3**	2(4.3±5.1)	8(6.9±4.4)	19.7±4.2
**P**	0.00*	0.00*	0.03*

Notes: * There are statistically significant differences (p <0.05).

**Table 4:** Knowledge and practice of the following preventive measurements among the studied participants.

-	**No/Rare** **F (%)**	**Sometimes /Often** **F (%)**	**Always** **F (%)**	**Total Practice Score**
**The practice of preventive measurements during the previous three weeks**				
**Hygienic hand wash with water and soup**	28(3.3)	210(24.5)	619(72.3)	Median20(mean±SD))19. ±3.9(Range9-27
**Hygienic hand wash with alcohol 70%**	219(25.6)	271(31.6)	361(42.8)	
**Safe distance (1-2m)**	83(9.7)	272(31.7)	503(58.6)	
**Surface sterilization**	213(24.9)	286(33.4)	358(41.8)	
**Wearing face masks**	259(30.2)	236(27.5)	362(42.2)	
**Sanitize clothes after return homes**	269(31.4)	253(29.5)	335(39.1)	
**Eat foods from outdoors**	736(85.6)	77(9.0)	441(39.1)	
**Sanitize purchases**	230(26.8)	261(30.5)	366(42.7)	
**Stay home**	58(6.8)	129(15.1)	670(78.2)	
**Knowledge of the practice of sneezing etiquette and hygienic hand wash.**				
	**No** **F (%)**	**Yes** **F (%)**	**Not sure** **F (%)**	
**Sneezing etiquette**	31(3.6)	809(94.4)	17(2.0)	
**Hygienic hand wash**	8(0.9)	832(97.1)	17(2.0)	

**Table 5:** Generalized Anxiety Disorder 7-item (GAD-7), and PHQ-9 (Patient Health Questionnaire-9 scale during the previous two weeks.

	Not at allF (%)	Several DaysF (%)	More than Half DaysF (%)	Nearly Every DayF (%)
**Generalized Anxiety Disorder 7-item (GAD-7)**				
**Feeling nervous, anxious, or on edge**	371(43.1)	371(43.1)	67(7.8)	52(6.0)
**Not being able to stop or control worrying**	523(60.7)	237(27.5)	57(6.6)	44(5.1)
**Worrying too much about different things**	573(66.6)	200(23.2)	49(5.7)	39(4.5)
**Trouble relaxing**	505(58.7)	258(30.0)	49(5.7)	49(5.7)
**Being so restless that it's hard to sit still**	654(76.0)	148(17.20	27(3.1)	32(3.7)
**Becoming easily annoyed or irritable**	426(49.5)	305(35.4)	64(7.4)	66(7.7)
**Feeling afraid as if something awful might happen**	556(64.6)	197(22.9)	55(6.4)	53(6.2)
**Total Score Median (mean±SD) (range)**	2(4.01±4.9)(0-21)			
**Degree of anxiety** **No** **Mild** **Moderate** **Severe**	F (%)260(30.2)355(41.2)162(18.8)84(9.8)			
**Patient Health Questionnaire-9(PHQ-9)**				
**Little interest or pleasure in doing things?**	281(32.6)	397(46.1)	86(10.0)	97(11.3)
**Feeling down, depressed, or hopeless?**	394(45.8)	324(37.6)	74(8.6)	69(8.0)
**Trouble falling or staying asleep, or sleeping too much?**	364(42.3)	315(36.6)	97(11.3)	85(9.9)
**Feeling tired or having little energy?**	291(33.8)	368(42.7)	102(11.8)	100(11.6)
**Poor appetite or overeating?**	413(48.0)	283(32.9)	92(10.7)	73(8.5)
**Feeling bad about yourself or that you are a failure or have let yourself or your family down?**	514(59.7)	235(27.3)	64(7.4)	48(5.6)
**Trouble concentrating on things, such as reading the newspaper or watching television?**	524(60.9)	206(23.9)	79(9.2)	52(6.0)
**Moving or speaking so slowly that other people could have noticed? Alternatively, so fidgety or restless that you have been moving a lot more than usual?**	493(57.3)	254(29.5)	68(7.9)	46(5.3)
**Thoughts that you would be better off dead, or thoughts of hurting yourself in some way?**	794(92.2)	37(4.3)	16(1.9)	14(1.6)
**Total score Median (mean±SD) (range)**	5(6.4±6.0) (0-27)			
**Degree of depression** **None** **Mild** **Moderate** **Moderately severe** **Severe**	F (%)128(14.9)281(32.6)257(29.4)104(12.1)50(5.8)41(4.8)			

**Table 6:** Correlation between the total practice score of preventive measurements and the following variables.

Total Scores	Depression / Practice	Anxiety / Depression	Anxiety / Practice
r(p)	-0.02(0.49)	0.79(0.00)*	0.01(0.73)

* There are statistically significant differences (p <0.05)

**Table 7:** Public response during the first wave of the COVID-19 pandemic.

-	NoF (%)	YesF (%)
**Growth zone**		
I practice quietude, patience, relationships and creativity	89(10.3)	772(89.7)
Looking for new ways to disease	80(9.3)	781(90.7)
I thank and appreciate others	42(4.2)	819(95.1)
Think about others and help them	51(5.9)	810(94.1)
Do your best to support others	79(9.2)	782(90.8)
**Learning zone**		
I evaluate information before spreading something false	91(10.6)	770(89.4)
I identify my emotions	223(25.9)	638(74.1)
I stop compulsively consuming what hurts me, from food to news	335(38.9)	526(61.1)
I start to give up what I can’t control	432(50.2)	429(49.8)
**Fear zone**		
I complain frequently	730(84.8)	131(15.2)
I spread emotions related to fear and anger	807(93.7)	54(6.3)
I grab food, toilet paper and medications that I don’t need	694(79.7)	177(20.3)

**Table 8:** Participants’ perception or feeling towards COVID-19 s during the first wave of the COVID-19 pandemic.

**Total**	**F (%)**
**Fear to infect my family**	647(75.1)
**Afraid because I’m the only source of income for my family**	48(5.6)
**The course of the disease in the hand of God**	626(72.7)
**A simple and not afraid**	105(12.2)
**I fear death**	139(16.1)
**Afraid of the lack of treatment opportunities and poor health care**	40(4.7)
**Afraid of the complication as I’m risky**	223(25.9)
**Other**	38(4.4)

**Table 9:** The Emotional Contagion Scale among inhabitants in KSA during the first wave of the COVID-19 pandemic.

-	NoF (%)	RareF (%)	SometimesF (%)	OftenF (%)	AlwaysF (%)	Total Scoremean±SD
**Sadness**						
**If someone I am talking with begins to cry, I get teary-eyed.**	19(2.2)	364(42.3)	112(13.0)	284(33.0)	82(9.5)	2.90±0.97
**I am filled with sorrow when people talk about the death of their loved ones.**	16(1.9)	112(13.0)	246(28.6)	255(29.6)	232(26.9)	3.72±1.01
**I cry at sad movies.**	17(2.0)	324(37.6)	256(29.7)	137(15.9)	127(14.8)	3.09±1.07
**Love**						
**When I look into the eyes of the one I love, my mind is filled with thoughts of romance**	8(0.9)	144(16.7)	307(35.7)	208(24.2)	194(22.5)	3.53±1.02
**I melt when the one I love holds me close.**	6(0.7)	47(5.5)	92(10.7)	213(24.7)	503(58.4)	4.37±0.88
**I sense my body responding when the one I love touches me**	9(1.0)	102(11.8)	166(19.3)	213(24.7)	371(43.1)	4±1.06
**Happiness**						
**Being with a happy person picks me up when I am feeling down.**	12(1.4)	106(12.3)	127(14.8)	294(34.1)	322(37.4)	3.98±1.05
**When someone smiles warmly at me, I smile back and feel warm inside**	6(0.7)	34(3.9)	93(10.8)	289(33.6)	439(51.0)	4.19±0.92
**Being around happy people fills my mind with happy thoughts.**	6(0.7)	53(6.2)	136(15.8)	259(30.1)	407(47.3)	4.33±0.82
**Fear**						
**Watching the fearful faces of victims on the news makes me try to imagine how they might be feeling.**	12(1.4)	244(288.3)	316(36.7)	182(21.1)	107(12.4)	3.37±0.99
**I notice myself getting tense when I am around people who are stressed out.**	8(0.9)	240(27.9)	349(40.5)	169(19.6)	95(11.0)	3.87±1.03
**Listening to the shrill screams of a terrified child in a dentist’s waiting room makes me feel nervous.**	12(1.4)	221(25.7)	253(29.4)	183(21.3)	19.2(22.3)	3.41±1.09
**Anger**						
**I clench my jaws and my shoulders get tight when I see the angry faces on the news.**	22(2.0)	530(61.6)	201(23.3)	72(8.4)	36(4.2)	3.18±0.99
**It irritates me to be around angry people.**	7(0.8)	106(12.3)	202(23.5)	248(28.8)	298(34.8)	2.54±0.83
**I tense when overhearing an angry quarrel.**	10(1.4)	178(20.7)	330(38.3)	197(22.9)	146(17.0)	3.14±0.95

## Data Availability

The data that support the findings of this study are available from the corresponding author, [S.A.A], upon reasonable request.

## LIST OF ABBREVIATIONS

SARS-CoV-2: Respiratory Syndrome Coronavirus-2
WHO: World Health Organization
KSA: Kingdome of Saudi Arabia
BC: British Colombia
EC Scale: Emotional Cognition Scale
GAD-7: Generalized Anxiety Disorder Assessment
PHQ-9: Patient Health Questionnaire-9 scale
UN: United Nations
